# The Guy’s and St Thomas’s NHS Foundation Trust @home service: an overview of a new service

**DOI:** 10.1080/17571472.2016.1211592

**Published:** 2016-07-29

**Authors:** Geraldine A. Lee, Karen Titchener

**Affiliations:** a Department of Adult Nursing, Florence Nightingale Faculty of Nursing & Midwifery, King’s College London, London, UK; b Guy’s and St Thomas’s Trust @home service, Walworth Clinic, London, UK

**Keywords:** Hospital in the home, community care, admission avoidance, early discharge

## Abstract

Hospital in the home is a relatively new concept within the UK healthcare system. The Guy’s and St Thomas’s NHS Foundation Trust (GSTT) @home service ‘Bringing hospital care to your home’ was commissioned by Lambeth and Southwark CCG in 2014 to provide acute care in the patients’ place of residence by facilitating rapid discharge from hospital. The service is designed for 260–280 referrals each month from local hospitals, London Ambulance Service, GPs, district nurses and palliative care services. The GSTT@home provides intensive care for a short episode through multidisciplinary team work with the aim of returning the patient to their prior health status following an acute episode of ill health. The main criteria for referrals are adults, living within Lambeth or Southwark with an acute onset of illness often with acute exacerbations of chronic conditions. Care is delivered using 25 clinical pathways using integrated care teams, including those for respiratory disease, heart failure and palliative care services. Recently, the service extended to include overnight palliative care. As care shifts from hospital to the community, it is envisaged that these types of programmes will become an essential component of care provision. This paper describes the service and presents initial service evaluation data.

## Why this matters to me

With the chronic shortage pressure on inpatient beds, there is a need to change the model of care. Treating people in their homes allows this with a multidisciplinary team. The GSTT@home team are able to provide care for a wide variety of conditions and provide many treatments and receive referrals from many sources. These sources include the local hospitals (allowing for early and safe discharge), from general practitioners (avoiding hospital admissions) and from others including London Ambulance Service (reducing the burden on emergency departments). The @home provides care seven days a week and has proven to be both efficient and cost-effective. It also highlights the ability to move acute care from acute settings to the community. We hope that this model of care can be extended to other areas in the future.

## Key message

Care for acute episodes can be provided in people’s homes using a multidisciplinary approach.

## Introduction

Hospital in the home is a relatively new concept within the UK healthcare system despite being an established method of delivering community based care in several countries.[1–5] This paper will provide an overview of the relatively new Guy’s and St Thomas’s NHS Foundation Trust (GSTT) @home and describe how the service has evolved since its inception.

### Hospital in the home literature

Within the NHS, the last few years has seen unprecedented demands on the service due to an ageing population and multiple co-morbidities. With 11 million people over 65 years and four million people with a long-term illness in the UK, many patients require regular healthcare in both acute and community settings.[6,7] With pressure on hospital beds, where appropriate emergency department (ED) staff try to discharge older patients with long-term illnesses back into the community for further management and avoid a scenario known as ‘bed or access block’ (i.e. lack of social support or inability to self-care at home).[8] A King’s Fund report noted the challenges of caring for older people and the complexity involved in assuring the availability of community services, access to hospital services and the relationships between the various services and their staff.[8] The report highlighted the need for better alignment of primary, community and acute care to reduce avoidable hospital admissions and length of hospital stays. One of the solutions to reduce hospital bed use is a ‘hospital in the home’ model that allows patients to return home and receive short-term treatment in a familiar environment.

Hospital in the Home (HitH) was first described in 1958, when it was proposed that some of the clinical interventions performed in hospital could be undertaken within a person’s home.[9] Since then, many countries have created their own version of hospital in the home services and reported very positive results.[1–5]

The aspects of HitH that have been evaluated include its safety, efficacy, patient satisfaction and cost. An early RCT examined the safety and patient/carer satisfaction comparing the effectiveness of treatment of acute illness at home and in hospital and found lower incidence of geriatric complications (in those over 65 years) including confusion, urinary and bowel complications in the HitH patient group compared to the hospital patient group (all *p* < .01).[1] Patient and carer satisfaction was also greater in the HitH group. A review of Australian HitH programmes 2008–09 recorded 32,462 admissions which represented 5% of all bed-days in the State of Victoria and a total of 26,653 bed days were delivered with these patients requiring on average nine visits from nurses and four from physicians.[3] Rodriguez-Cerrillo et al. [4] reported that HitH yielded a decrease in unscheduled returns to hospital from 7 to 3%. Varney et al.’s integrative literature review of HitH programmes concluded that HitH care was at least equivalent to hospital-based care and offered greater cost savings and should be expanded.[5]

Overall, the literature reports the positive impact of HitH upon patient care,[10] although the issue of implementing knowledge management practices has been raised by some.[11] These authors highlighted the importance of maintaining and applying evidence to clinical practice but conceded the difficulties associated with it. Nonetheless, the overall efficacy of the HitH programme has been consistently demonstrated.[1,3,5,10]

## Methods

### The Guy’s and St Thomas’s NHS foundation trust @home service

The Guy’s and St Thomas’s NHS FoundationTrust (GSTT) @home service ‘Bringing hospital care to your home’ was commissioned in 2013 by Lambeth and Southwark CCG to provide acute inpatient care in the patients’ place of residence for adults over 18 years of age. Its primary aim was to facilitate the rapid discharge from accident and emergency departments, acute assessment units and acute wards by allowing patients to be discharged from hospital earlier (early discharge) or avoiding patients being admitted to hospital (admission avoidance). See Table 1 for its key characteristics.

**Table 1.** Key characteristics of the GSTT@home service.

(1) It provides care to all adults in their usual place of residence who need acute care at home but who are not necessarily housebound. (2) It mainly employs senior nurses with acute hospital nursing experience in Emergency Departments, Intensive Care Units and medical assessment wards, who are skilled at managing complex acutely unwell patients. Nurses are usually educated to Masters Level and also have advanced differential diagnostic skills and are non-medical prescribers. (3) It provides intensive care for a short episode through multidisciplinary team work with the aim to return the patient to their prior health status following an acute episode of ill-health. (4) The service will see a patient within two hours of referral for a maximum of seven days usually. (5) It offers overnight palliative care (pal@home), with a rapid response ‘out of hours’ urgent/crisis nursing care service. pal@home provides prompt clinical support and nursing care at short notice, through proactive visits, or in response to an unscheduled request. It is for patients who are identified as end-of-life, are nearing death or require palliative or @home out-of-hospital support and who meet the service referral criteria.

### The service

The service is commissioned to receive referrals from three main sources: GPs, St Thomas’ Hospital (STH) and King’s College Hospital (KCH). GSTT@home has a clinical lead nurse with experience of establishing new services, who leads and develops the team both strategically and operationally. It also has consultant geriatrician support from both STH and KCH who contribute to the multidisciplinary team (MDT), will visit patients in their own homes or see them urgently in their clinics. The teams comprise senior nurses led by matrons and each team also has GPs, rehabilitation support workers, physiotherapists, occupational therapist, social workers and a pharmacist. The MDT assesses, initiates and implements treatment, and meets daily to discuss the progress of the patient. Due to the acuity of the patients, most nurses are bands 6 and 7 (supported by a few band 5 nurses). The service also offers placements to student nurses who have given very positive feedback on their experiences.[12]

GSTT@home service brochures have been created for patients and healthcare professionals and highlight the collaborative nature of the team in working with GPs, hospital staff and other organisations to deliver safe and high quality healthcare within the patient’s own home. The staff brochure sets out three specific aims:

(1) Identifying people at risk of a hospital admission and providing care that prevents their condition from worsening;
(2) allowing people to be given a high level of care in their own homes instead of being admitted unnecessarily to hospital and;
(3) allowing for advanced discharge from hospital so that patients can recuperate in the comfort of their home while receiving high quality care.

**Figure 1.**
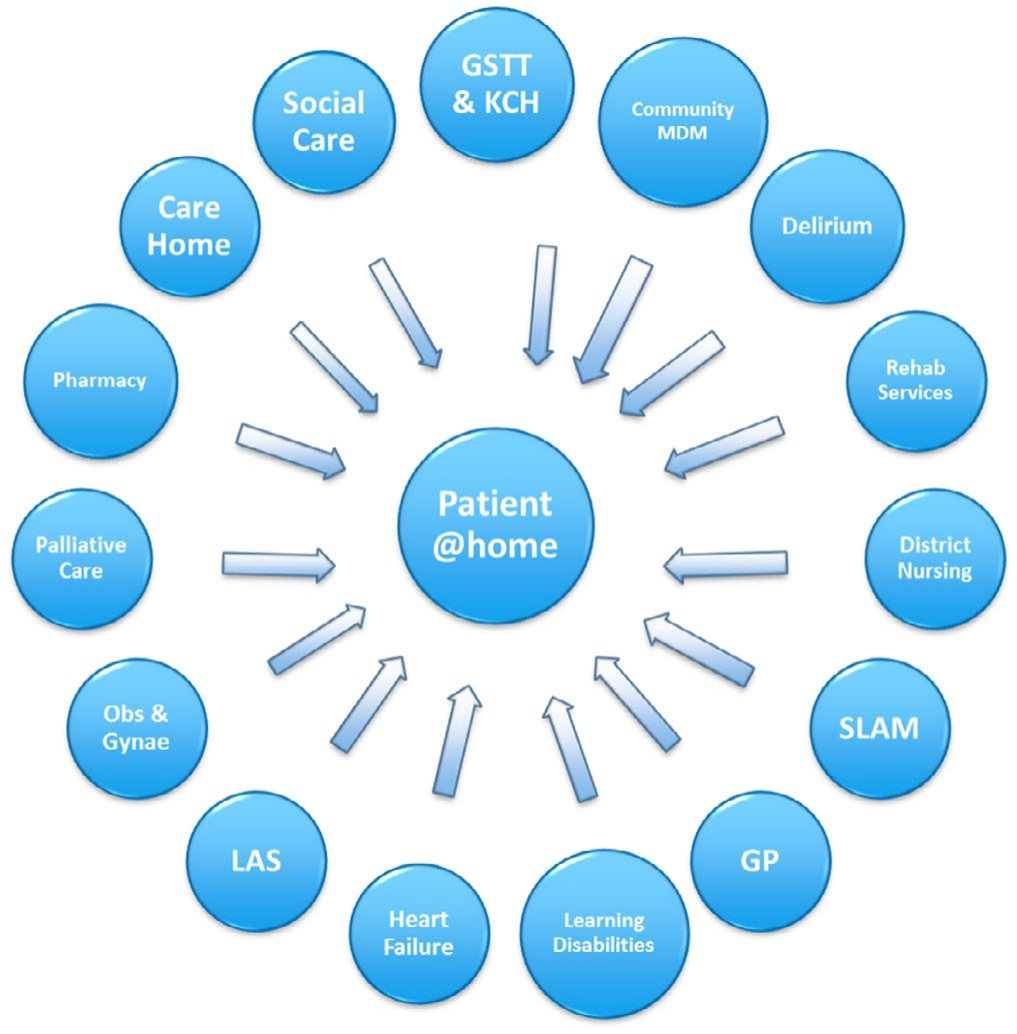
Integrated partners with GSTT@home.

The service is designed for 260–280 referrals each month that can be made by medical, nursing and therapy staff from the acute hospitals as well as GPs, community nurse specialists (such as heart failure nurses, community matrons or specialist palliative care), emergency departments and London Ambulance Service staff. The service operates 24/7 with the overnight service mainly focused on palliative and end-of-life care or an acute medical emergency such as blocked urinary catheter. The service receives around 20 referrals daily. The main referral criteria are: adults aged 18 years and over, living within Lambeth or Southwark with an acute onset of illness (these may include acute exacerbations of chronic conditions). Most patients are either: early discharge (following a medical procedure in hospital and requiring further nursing care or therapy) or admission avoidance (the patient has been identified as being at high risk of requiring a hospital admission). All referrals are triaged by the GSTT@home duty clinician (matron or GP) or, if they are inpatients, they are reviewed and assessed by a GSTT@home in-reach nurse. The in-reach nurses in both hospitals (STH and KCH) review patients in the emergency departments, acute assessment wards and on post-take rounds identify suitable patients as quickly as possible. The GSTT@home duty clinician or in-reach nurse determine if the referral is appropriate and fits the acceptance criteria (i.e. requires short-term care in their own home that can be provided by the team). The patient is then transferred to the appropriate team where they will be assessed initially by a senior nurse or GP. All patients’ GPs are informed that the patient has been seen by the GSTT@home team and will be sent an intervention summary on discharge from the service. As can be seen, there are many other referral sources, clinical pathways and partnerships (currently around 25) (see Figure 1).

The @home team offers care for many conditions and have developed a range of pathways including those for hyperemesis gravidarum, respiratory conditions, heart failure, learning disabilities, care home residents with/out dementia. The GSTT@home team clinical interventions are set out in Table 2.

**Table 2.** Clinical interventions offered by the GSTT@home team.

Rapid assessment, diagnosis, treatment and evaluation
Medication titration
Intravenous/subcutaneous fluids
Intravenous antibiotics
Intravenous diuretics
Treatment for respiratory disorders including nebulisers, antibiotics, physiotherapy
Bladder scans for post-gynaecological surgery
Trial without catheter (post-operatively)

Table 3 sets out the patient conditions which have been managed with requests for support for additional conditions carefully considered on a case by case basis to ensure they fit the GSTT@home team remit. Referrers are required to provide the following information: current medication and any newly prescribed medications, the presence of any invasive vascular device such as a PICC line, any infectious condition, recent investigations, if under a hospital consultant, their details and last communication letter and past medical history. Using clear criteria ensures transparency and clarity in the referral process.

**Table 3.** Possible patient conditions that the GSTT@home team can manage.

Cellulitis	Falls
Chronic obstructive pulmonary disease	Unstable Diabetes
Dehydration	Palliative Care
Gastroenteritis	Community Acquired Pneumonia
Heart failure	Deep Vein Thrombosis
Infected foot ulcers	Hyperemesis Gravidarum
Post-operative surgery	Pyelonephritis
Urinary tract infection	Viral Illness

## Results

Care protocols have been developed to deliver safe care based on the best available evidence. As can be seen in Table 4, the monthly referrals have increased over 2015; 263 in September, 275 in October, 265 in November and 348 in December (when the service commenced 24/7 care). In January and February 2016, 405 and 385 patients were referred respectively. The main reasons for referral were: COPD exacerbation (22%), IV therapy (including IV antibiotics, diuretics and fluids) (23%), heart failure (16%), urinary tract infections (13%), falls (5%), community acquired pneumonia 5%, diabetes 2% and blood monitoring for acute kidney infection and INR, 10%. The average length of GSTT@home stay is around 7 days with readmissions to acute hospitals around 17% a month and readmission to GSTT@home around 14% a month of the total accepted referrals.

**Table 4.** Month-on-month received and accepted referrals from April 2015 to March 2016.

Months	Apr-15	May-15	Jun-15	Jul-15	Aug-15	Sep-15	Oct-15	Nov-15	Dec-15	Jan-16	Feb-16	Mar-16
Received referrals	339	323	418	405	355	378	399	288	356	375	359	358
Accepted referrals	264	267	343	311	242	263	275	200	276	279	260	282
% Accepted/ Received referrals	78	83	82	77	68	70	69	69	78	74	72	79
GSTT In-patients	85	66	84	79	60	70	70	57	23	67	62	74
GSTT A&E	8	4	3	3	3	5	4	2	2	5	5	4
KCH In-patients	54	50	73	55	44	51	67	36	51	80	59	62
KCH A&E	8	11	41	22	14	13	14	15	2	2	3	3
Total	155	131	201	159	121	139	155	110	78	154	129	143
% Accepted referrals	59	49	59	51	50	53	56	55	28	55	50	51
GP	56	67	69	76	56	57	56	41	97	61	66	66
Community services	15	23	20	21	22	17	20	19	42	24	26	31
London ambulance service	37	46	44	54	42	50	44	30	59	40	39	42
Total	108	136	133	151	120	124	120	90	198	125	131	139
% Accepted referrals	41	51	39	49	50	47	44	45	72	45	50	49

The initial evaluation indicates that the service is meeting its aims with positive feedback from both patients and their relatives and attainment of clinical outcomes. The clinical benefits include effective and efficient integrated partnerships, a reduction in emergency department attendances, a reduction in length of hospital stays and associated costs, reduced conveyance times allowing the ambulance service to go to the next emergency call and a reduction in inappropriate hospital admissions, reduced risk of hospital acquired infections and reduced delirium and confusion. From the patient perspective, the benefits include improved health outcomes, a preference for being treated at home rather than in hospital, reduced pain and anxiety and the psychological and social benefits of being treated in their own home.

## Discussion

This GSTT@home service was able to care for a wide range of conditions including COPD, dehydration, infected foot ulcers and diabetes. Managing chronic diseases is a major part of hospital based care and places an enormous burden on the system. Being able to manage acute episodes of chronic conditions in the community with intravenous antibiotics and fluids has previously been reported as cost effective and safe.[1,3,5,10] The use of the MDT is an essential part of the service and reduces the need for hospital admission or can reduce length-of-stay. Robertson et al. [13] highlighted case studies where complex patients were treated at home and in primary care although most focussed on single conditions such as dermatology and respiratory services. The shift from specialist care to community care can be done but requires a strong MDT team and a redesign of existing pathways.[13,14]

A thorough evaluation of the GSTT@home programme is needed to assess its effectiveness and appropriateness and to gather more information on its safety, patient and staff satisfaction and financial benefits. Others have clearly demonstrated the benefits and propose that hospitals invest in HitH medical leadership and supervision to expand their HitH services, including teaching.[3] Unfortunately only limited data are available on complications and readmissions from HitH programmes. Montalto et al. recorded a 4% unplanned return to hospital (*n* = 143) with an unexpected mortality rate of 0.15%; these patients were aged over 50 years and had received intravenous antibiotic therapy.[10] Their findings reflect ‘real-life’ scenarios rather than a controlled trial with data collected from 2000 to 2007. The use of protocols in providing safe care is not described in the literature with the exception of Rodriguez-Cerrillo et al. who noted high compliance rates of 97%.[4] Use of evidence based and clinically relevant protocols should be the cornerstone of any HitH programmes.

Undoubtedly, efficient treatment of patients in their home is a worthy goal, but it is important that the appropriate healthcare professionals provide the care and work together to ensure that it is achieved. For example, the GSTT@home team will refer to other healthcare professionals such as community-based physiotherapists and occupational therapists as required. This fits well with Robertson et al. who suggested the use of consultant-run email and telephone helplines and outreach clinics that are jointly staffed by hospital consultants and other healthcare professionals.[13] A major change in care delivery using advanced practice will require upskilling of the non-medical workforce.[15] There are many examples of this happening via the NHS vanguards with new models of care that are integrated and delivered in the community using an MDT approach.[16] Care post-hospital discharge has changed significantly over the past two decades [17] and we plan to undertake detailed analyses of patient outcomes together with the clinical and financial benefits of the GSTT@home service.

## Conclusion

The growing number of older people presents a challenging healthcare burden with over 20 million people aged over 60 years expected by 2030.[6] As care shifts from hospital settings to the community, programmes such as the GSTT@home service will become an essential component of health care provision. A more detailed evaluation of the GSTT@home service would be useful to understand its contribution to the local health system and inform ongoing service development.

## Conflicts of interest

The authors report no declarations of interest.
